# Global Rise of Compounded Weight-Loss Medicines: A Worrisome Trend

**DOI:** 10.1210/jendso/bvaf084

**Published:** 2025-06-07

**Authors:** Nikhil Sood, Rohini Garg

**Affiliations:** Department of Hospital Medicine, Banner Gateway Medical Center, Banner Health, Gilbert, AZ 85034, USA; Department of Internal Medicine, CHI Health Mercy Hospital, Council Bluffs, IA 51503, USA

**Keywords:** compounded drugs, weight loss drugs, semaglutide, tirzepatide

## Abstract

The rise of compounded weight-loss drugs has emerged as a pressing global health issue, threatening consumers and health-care systems. Factors fueling this crisis include the desire for rapid weight-loss options, inadequate regulatory frameworks, and the easy availability of compounded products through online platforms and social media. Vulnerable populations, particularly those searching for low-cost treatments, are hit hardest. Tackling this escalating problem calls for a comprehensive strategy: enhancing regulatory oversight, raising public awareness, and fostering stakeholder collaboration. We can reduce the health dangers of compounded weight-loss medications by addressing the underlying causes and improving consumer safety. This article examines the risks associated with compounded weight-loss drugs and suggests practical measures to protect public health.

## Background

Semaglutide, tirzepatide, and liraglutide are the 3 gut-hormone-based drugs that are glucagon-like peptide-1 (GLP-1) and glucose-dependent insulinotropic polypeptide (GIP/GLP-1) agonists currently approved by the US Food and Drug Administration (FDA) for chronic weight management [1, 2]. The surging demand for these drugs has far outpaced supply, fueling global supply problems and chronic shortages, which have led consumers to turn to counterfeit and compounded versions of these drugs and online pharmacies [3, 4]. A drug shortage declared by the FDA enables compounding pharmacies to use a regulatory exception to replicate the brand-name drugs for dispensing to consumers under the direction of their prescriber. Tirzepatide and semaglutide have been in shortage since 2022 due to increased demand, but recently, the FDA has determined that the shortage has been resolved [5, 6]. The FDA has given up to 3 months to compounding pharmacies to stop the production of these medications while the global supply chain is being restored [7]. Meanwhile, the association of compounders has sued the FDA, and the matter is in court. We aim to explore the global issue of compounded weight-loss medications and discuss ways to tackle this public health concern. We also briefly discuss online pharmacies, emphasizing the increasing worries associated with this.

## Compounded Drugs

Drug compounding is often regarded as combining, mixing, or altering ingredients to create a medication tailored to a patient's needs [8]. The FDA allows for compounding of FDA-approved products listed on the FDA shortage list only to facilitate continuity of treatment and uninterrupted access to prescribed pharmacotherapies [9]. Human drug compounding is regulated per sections 503A and 503B of the Federal Food, Drug, and Cosmetic Act (FD&C Act) [10]. Section 503A applies to human drug compounding by a licensed pharmacist within a state-licensed pharmacy or federal facility or by a licensed physician. In contrast, section 503B guides human drug compounding by outsourcing compounding facilities [10]. Compounding under section 503A allows the production of relatively small quantities of compounded products, with 503A compounding facilities primarily regulated by state boards of pharmacy. In contrast, 503B facilities are registered with and are inspected by the FDA [10]. These facilities can manufacture large quantities of compounded products, but only while these products are actively listed on the FDA shortage list [9]. The shortage of GLP-1 drugs enables compounding pharmacies to use a regulatory exception to replicate the brand-name drugs for dispensing to consumers by purchasing active pharmaceutical ingredients from FDA-registered manufacturers [8].

## Concern With the Use of Compounded Glucagon-like Peptide-1 Agonists

Compounded drugs have been found to have more significant risks than FDA-approved drugs as there is less oversight, and they are not clinically tested for safety or efficacy [11]. As of November 30, 2024, the FDA has received 392 reports of adverse events with compounded semaglutide and 215 reports with compounded tirzepatide [12]. The American Diabetes Association recently issued a statement recommending against using non–FDA-approved compounded GLP-1 and dual GIP/GLP-1 receptor agonist products due to safety, quality, effectiveness concerns, and uncertainty about their content [13]. Data on the adverse consequences of compounded weight loss medication use are only beginning to emerge.

Concerns regarding compounded GLP-1 receptor agonists stem from insufficient data on their bioequivalence, biodistribution, and elimination relative to FDA-approved formulations [8]. Compounded products may use different salt forms, excipients, or delivery systems, affecting absorption, peak plasma concentrations, and therapeutic outcomes [14]. Biodistribution, or how the drug disperses throughout the body, may also differ. Modifying formulation can affect tissue penetration, half-life, and receptor engagement, particularly in long-acting GLP-1 agents, for which sustained activity is critical [8]. For example, particle size or solubility changes can alter how the drug reaches peripheral tissues or the central nervous system. Whether these compounded versions achieve comparable efficacy or safety profiles without rigorous pharmacokinetic testing is unclear.

Nonstandardized product labeling of compounded drugs and the lack of standard instructions regarding contraindications, warnings, etc, can lead to dosing errors [12]. Incorrect dosage may also result from differing concentrations of the same drug in different compounded formulations or incorrect use of syringes [12]. Some compounders incorporate additional ingredients, such as cyanocobalamin (vitamin B12), pyridoxine (vitamin B6), levocarnitine (L-carnitine), and nicotinamide adenine dinucleotide (NAD), into their semaglutide products [11]. Some compounding pharmacies use salt forms of semaglutide, including semaglutide sodium and semaglutide acetate [11]. The salt forms are different active ingredients from those used in the approved drugs, which contain the base form of semaglutide. The safety and effectiveness of combining semaglutide with other ingredients have yet to be established. This can lead to mismanagement of blood glucose, body weight, and other side effects. The FDA recommends not using salt forms to compound semaglutide [11].

Most of the time, compounding pharmacies compound drugs to meet the needs of patients who cannot use an approved drug. However, during a drug shortage, outsourcing facilities may compound drugs that are identical or nearly identical to FDA-approved drugs on the FDA drug shortages list. Three cases of adverse drug events related to the incorrect administration of semaglutide were reported after the drug was obtained from compounding pharmacies and an aesthetic spa [14]. Lilly, the parent company, expressed concern about the increasing reports of off-label use of tirzepatide and the risk associated with tirzepatide products not approved by the FDA [15].

## Ongoing Legal Battle

The ongoing dispute between the FDA and compounding pharmacies over GLP-1 receptor agonists is causing considerable uncertainty for patients who depend on these drugs for managing weight and treating diabetes. As a consequence of this conflict, it is unclear how the shortage will affect patients, posing challenges for patients to acquire the necessary treatments. Although the FDA does not approve compounded versions, some patients choose them for cost or availability. Legal ambiguity around compounded drugs forces patients to face higher costs or longer wait times for FDA-approved therapies. Many may choose compounded options, lacking FDA-approved drugs’ rigorous safety and efficacy testing. Ultimately, this legal dispute limits access to transformative treatments, delays critical care, and creates confusion about the safety of alternatives.

## Online Pharmacies

The substantial growth of online pharmacies has exacerbated illegal sales of weight-loss medications outside the regulated drug supply system [16]. These pharmacies offer easy access, deep discounts, and lower prices than legitimate pharmacies, but the drug may be potentially adulterated, misbranded, or contain dangerous ingredients [17]. The shortage, exponential demand, and the fact that injectable weight-loss drugs are often not covered by insurance drive the pivot toward these pharmacies. When patients purchase medications from illegal online sellers, they frequently do not fully understand the risks of taking the illegally sold drugs. Many illegal online pharmacies advertise the sale of semaglutide injections without requiring a prescription [18]. On September 30, 2024, the US Department of Justice announced an indictment against individuals running illegal online pharmacies [19].

## Critical Issues Driving the Problem

Critical issues driving the proliferation of compounded weight-loss medications include the demand for quick-fix solutions, weak regulatory frameworks in many regions, unregulated online marketplaces, lack of ingredient transparency, and affordability concerns. We discuss some of these issues next.

1. Accessibility through online platforms: Numerous compounded weight-loss drugs are marketed on online platforms and social media, posing as legitimate brands [17, 18]. For instance, sellers frequently replicate the packaging of well-known weight-loss medications, making it difficult to tell them apart. Furthermore, the absence of regulation in online marketplaces worsens this issue, as these platforms seldom check the authenticity of third-party vendors’ health products [18].
2. Lack of transparency and vulnerability of consumers: Numerous consumers, particularly those suffering from obesity-related health issues, are eager for rapid solutions. This eagerness is exploited by promoting unverified products with deceptive assertions. For instance, a 2023 European investigation found weight-loss gummies containing sibutramine disguised as “herbal extracts.”
3. Effect on marginalized populations: Vulnerable populations that cannot afford legitimate weight loss treatments are disproportionately affected [20]. A lot of these compounded products are marketed at lower prices, attracting those with fewer resources and leading to worse health outcomes in already at-risk communities.
4. Lack of insurance coverage: Coverage for weight-loss medications such as Wegovy (semaglutide) or Zepbound (tirzepatide) varies by insurance plan. Many insurance providers may not cover prescription medications used strictly for weight loss. The lack of insurance coverage for weight-loss medications forces patients to seek compounded alternatives.

## Strategies to Address Issues With Compounded Weight-Loss Medications

The rising demand for weight-loss medications, particularly compounded versions, has created safety and regulatory challenges. While compounding can provide access to medications during shortages, inconsistent formulations, contamination risks, and misleading marketing have raised concerns. Addressing these issues requires targeted regulatory action, stricter quality control, and better public awareness.

1. Strengthening regulatory oversight
  - Clarify and enforce FDA guidelines for compounded weight loss medications—Certain pharmacies take advantage of regulatory loopholes to produce compounded forms of FDA-approved medications with insufficient oversight. Regulatory agencies must implement strict guidelines that restrict compounding to instances when an FDA-approved alternative is unavailable, unless there are verified shortages.
  - Enhance inspections of compounding pharmacies—Many of these facilities follow less stringent manufacturing standards than pharmaceutical companies. By increasing the frequency of inspections, we can better guarantee sterility, proper ingredient sourcing, and accurate dosing.
  - Mandate adverse event reporting—It is crucial for patients and health-care providers to have a formal process for reporting negative side effects related to compounded weight-loss medications, thus improving the collection of risk data on these products.
2. Improving quality control in compounded medications
  - Prohibit the use of unauthorized ingredient variations—Some compounded formulations substitute the FDA-approved semaglutide base with different salts, such as semaglutide sodium or acetate. This substitution could affect absorption and safety. Regulations need to clearly state that only bioequivalent ingredients are allowed.
  - Require third-party testing for potency and purity—Compounded medications should be subjected to independent testing to verify proper dosages and identify any contamination, safeguarding patient health.
  - Establish stricter sterility standards for injectable products. Since compounded injectables may not be prepared in sterile environments, regular sterility testing is essential to reduce the risk of infection.
3. Regulating online and telehealth sales
  - Monitor telehealth clinics that prescribe compounded weight-loss medications—Many online providers dispense these medications without sufficient medical oversight, increasing the risk of abuse. Licensing boards need to evaluate and oversee telehealth compounding services.
  - Limit misleading advertising on social media—Different influencers and online vendors promote compounded semaglutide and tirzepatide as cost-effective choices, often omitting possible risks. It is essential for social media platforms to enforce stricter advertising regulations for compounded drugs.
4. Educating health-care providers on safe prescribing
  - Train health-care professionals to recognize adverse effects—Compounded formulations can lead to unforeseen side effects from potency fluctuations or contamination. Providers need to be prepared to recognize and report these complications.
  - Promote FDA-approved alternatives and financial assistance programs—Patients often seek compounded medications for cost savings. Health-care professionals should direct them to copay assistance programs or manufacturer discounts before making recommendations.
5. Protecting consumers through transparency and education
  - Mandate full disclosure on compounded medications**—**Patients frequently obtain compounded medications without being made aware that these formulations lack FDA approval. Pharmacies must be mandated to offer both written and verbal disclosures when providing compounded weight loss drugs.
  - Create a publicly accessible database of accredited compounding pharmacies—A verified list of reputable compounding pharmacies should be kept, assisting consumers in identifying legitimate sources.
  - Launch public awareness initiatives on the risks of compounded medications—Educational campaigns should highlight potential risks, including potency inconsistencies and contamination concerns of compounded drugs.

## Conclusion

Compounded weight-loss medications present safety and regulatory challenges due to inconsistent formulations and inadequate oversight. Strengthening regulations, enforcing quality control, regulating online sales, educating health-care providers, and increasing consumer awareness are key steps in ensuring that compounded medications remain a safe and ethical option for patients who genuinely need them.

## Data Availability

Not applicable.

## Abbreviations

FDA: US Food and Drug Administration
GIP: glucose-dependent insulinotropic polypeptide
GLP-1: glucagon-like peptide-1
